# Clarifications for Variables and Findings and Corrections to Figures

**DOI:** 10.1001/jamanetworkopen.2023.28472

**Published:** 2023-08-08

In the Original Investigation, “Association of Video Gaming With Cognitive Performance Among Children,”^1^ published in *JAMA Network Open* on October 24, 2022, and retracted and replaced on April 10, 2023,^2^ additional clarifications for the variables and findings were needed. This includes clarification that there were differences in the unadjusted measures of body mass index and IQ between the video gamers and non–video gamers and that there were no such differences after adjusting for sociodemographic variables; similar clarifications to the relevant statistical analyses have been provided. The findings were clarified to acknowledge that the reaction times were measures of cognitive performance involving response inhibition and working memory and that the differences in reaction times between the 2 groups were very small. The original Table 1 has been corrected to report the unadjusted variables separately from the adjusted variables and outcomes, which are now reported in Table 2. The original Table 2 is now Table 3. Findings of the analyses reported in Supplement 1 have been clarified in the Results section of the main text, and a summary of additional analysis to address missing or partially missing data on the screen time questionnaire for 11 non–video gamer participants (0.5% of the sample) has been added to the Results section. In addition, errors were introduced by the journal to Figures 1 and 2. Markers and text labels were omitted from Figure 1. For Figure 2, the panel for the Child Behavior Checklist (CBCL) *t* scores was mislabeled as the CBCL raw scores, and a footnote indicator, a panel for the CBCL raw scores, and a dashed line for the CBCL *t* scores were omitted. The article has been corrected.^1^
