# Supplementary material for: Association of Video Gaming With Cognitive Performance Among Children
Source: JAMA Netw Open. 2022 Oct 24;5(10):e2235721. doi: 10.1001/jamanetworkopen.2022.35721 (PMC9593235; doi:10.1001/jamanetworkopen.2022.35721)
Supplement: Supplement 1. — eMethods. Stop Signal Task (SST), n-back Task, and fMRI Acquisition and Preprocessing eMethods and eResults. List Sorting and Rey Auditory Verbal Learning Test [file jamanetwopen-e2235721-s001.pdf]

Supplemental Online Content

Chaarani B, Ortigara J, Yuan DK, Loso H, Potter A, Garavan HP. Association of video gaming with cognitive performance among children. *JAMA Netw Open*. 2022;5(10):e2235721. doi:10.1001/jamanetworkopen.2022.35721

eMethods. Stop Signal Task (SST), n-back Task, and fMRI Acquisition and Preprocessing

eMethods and eResults. List Sorting and Rey Auditory Verbal Learning Test

This supplemental material has been provided by the authors to give readers additional information about their work.

© 2023 Chaarani B et al. *JAMA Network Open.*

eMethods. Stop Signal Task (SST), n-back Task, and fMRI Acquisition and Preprocessing

Stop Signal Task (SST)

The SST [23] adopted an event-related design and presented leftward and rightward facing arrows in serial order ("go" stimuli). Participants indicated the direction of the arrows using a two-button response box (left and right buttons). Participants were instructed to respond as quickly and accurately as possible but were told not to respond on trials in which a left or right arrow was followed by an arrow pointing upward (the "Stop" signal). The SST had two runs. Each had 180 trials, of which 30 were "Stop" trials, yielding a total of 60 "Stop" trials and 300 "go" trials. Each trial lasted 1sec. The time between the "go" and "stop" signals (the Stop-Signal Delay; SSD) varied dynamically based on a participant's success on the prior trial so as to achieve a 50% success rate (starting at 50 msec, the SSD increased by 50 msec if the participant successfully stopped on the previous trial, and decreased by 50 msec if he/she responded; see Figure 2-a). The main contrasts of interest probing inhibitory control are correct Stop vs. correct go and incorrect Stop vs. correct go.

Poor performance on the SST leading to exclusion was determined by: fewer than 150 go trials, less than 60% correct on go trials, incorrect go trials greater than 30%, late go trials (across correct and incorrect trials) greater than 30%, no response on go trials greater than 30%, fewer than 30 Stop trials, and Stop trial accuracy lower than 20% or greater than 80%. Children with SSRT less than 50 msec were excluded from the analysis (n=3).

N-back Task

In this working memory block-design task, participants saw a series of stimuli and indicated whether each one was the same or different than the stimulus *N* items earlier (i.e., "*N* back"). The n-back task included two conditions: a 2-back as the active condition and a 0-back as the baseline condition, which included similar visuo-motor demands but lower working memory load. In the 0-back condition, participants indicated if each stimulus matched a single target presented at the beginning of the trial, thereby obviating the need to maintain and update a two-item working memory load throughout the task. Responses on the 2-back and 0-back were input on a two-button keypad, with one button indicating the stimulus was a match and the other

© 2023 Chaarani B et al. *JAMA Network Open.*

indicating no match (see Figure 2-b). The n-back consisted of two runs, each containing eight blocks of trials and four 15 sec rest periods containing just a fixation cross. Blocks contained 10 trials lasting 2.5 sec each and were preceded by a 2.5 sec instruction screen indicating the condition for the upcoming block. Of the 10 trials in each block, 2 were targets, 2–3 were non-target lures, and the remainder were non-lures (i.e., stimuli only presented once). There were 160 trials in total with 96 unique stimuli of 4 different stimulus types (24 unique stimuli per type). Three-quarters of the stimuli types were human faces, demonstrating happy, fearful, or neutral facial expressions, with facial expression stimulus type held constant within each block. Faces were racially diverse and derived from two pre-existing collections: the NimStim emotional stimulus set [47] and the Racially Diverse Affective Expressions (RADIATE) set of stimuli [48]. For the working memory component, the main contrasts are 0-back vs. fixation, 2-back vs. fixation, and 2-back vs. 0-back (8 blocks each). Children with D'<0 (performing at chance-level) were excluded from the analyses (less than 1% of the sample).

fMRI Acquisition and Preprocessing

The full details of the imaging acquisition protocol were previously described in Casey *et al.* and Hagler *et al.* [11,21]. The ABCD Data Analysis, Informatics, & Resource Center (DAIRC) performed centralized initial quality control and processing of the fMRI data. All MRI assessments were reviewed by a neuroradiologist for incidental findings. Using a combination of automated and manual methods, the fMRI datasets were quality controlled for problems such as acquisition protocol compliance, imaging artifacts, motion or file corruption. Processing steps subsequent to fMRI preprocessing include the removal of initial frames to ensure equilibration of the T_1w signal and normalization of voxel time series by dividing by the mean across time of each voxel. The fMRI preprocessing pipeline started with a within-volume head motion estimation and correction by computing rigid body transformations between the first time point and each subsequent one. Scans were further processed for image distortions resulting from B0 field inhomogeneity, within voxel field gradients and gradient nonlinearities. 2.4 mm isotropic resampling was performed in order to align fMRI volumes across each participant, and a registration matrix computed with the T1w image. Estimates of task-related activation strength were computed at the individual subject level using a general linear model implemented in AFNI's 3dDeconvolve (Cox, 1996), which applied a general linear model (GLM) to each voxel's

© 2023 Chaarani B et al. *JAMA Network Open.*

time-series with additional nuisance regressors and motion estimates. Hemodynamic response functions were modelled with two parameters using a gamma variate basis function plus its temporal derivative (using AFNI's 'SPMG' option within 3dDeconvolve). Fast oscillatory signals within the motion estimates related to respiration, between 0.31 to .043 Hz, were temporally filtered with an infinite impulse response filter. Framewise displacement (FD) was then calculated from the filtered motion estimates, and frames with FD > .9 mm were censored. Preprocessed time courses were sampled onto the cortical surface for each individual participant, and then registered to the standard FreeSurfer surface atlas (fsaverage). After projecting to the surface, the data were smoothed along the cortical surface (5 mm). Voxels containing cortical gray matter were projected onto the surface by sampling values 1 mm from the gray/white boundary, into cortical gray matter, at each vertex (using FreeSurfer's mri_vol2surf with "white" surface, "-projdist 1" option, and default "nearest" interpolation). Average beta coefficients and standard errors were then computed for each of the two runs of each task and for each participant, weighted by the nominal degrees of freedom (number of frames remaining after motion censoring minus number of model parameters). Data used in the current study were derived from the data included in the ABCD data release 2.0.1, and included general linear model (GLM) beta coefficients and standard errors of the mean (SEM; calculated from the ratio of the beta and *t* statistic) calculated for each voxel and vertex. The full details of the task fMRI quality control and processing pipelines were previously described [11,21].

eMethods and eResults. List Sorting and Rey Auditory Verbal Learning Test

<u>Videogaming association with cognitive performance assessed with tasks not relying on visuo-motor coordination</u>

Performing the Stop Signal and EN-back fMRI tasks involves eye-finger coordination since it requires participants to watch changing stimuli from the in-scanner screen and respond to those stimuli by pushing buttons on a handheld device. Videogaming, particularly on consoles and computers, involves a similar visuo-motor process. This may introduce a confound since VG in the fMRI samples may have a significant "practice effect" advantage in terms of eye-finger coordination compared to NVG. To investigate this, we carried out linear mixed models

© 2023 Chaarani B et al. *JAMA Network Open.*

comparing cognitive measures between VG and NVG from two tasks that do not involve eye-finger coordination:

- A list sorting working memory task included in the Youth National Institutes of Health (NIH) Toolbox®, a sequencing task requiring children to sort and sequence stimuli that are presented visually and auditorily. A list sorting score for each participant was computed and used in the analysis.
- The Rey Auditory Verbal Learning Test (RAVLT), which is widely used to evaluate verbal learning and memory, including proactive inhibition, retroactive inhibition, retention, encoding versus retrieval, and subjective organization. An accuracy score was derived for each participant and used in the analysis.

Our analyses revealed that VG scored lower on the list sorting task than NVG (adjusted mean: 94.1 vs 95.8; $P = 0.017$), while the accuracy on the RAVLT was not different between VG and NVG (adjusted mean 8.8 vs 8.9; $P=0.74$). There were also no differences between the sexes regarding the association between videogaming and cognition measures. Although these tests are inconclusive and do not perfectly mimic the in-scanner inhibitory control and emotional working memory tasks, these out-of-scanner results do suggest that the performance superiority associated with videogaming could be partially due to better visual-motor coordination.

© 2023 Chaarani B et al. *JAMA Network Open.*
