# Supplementary material for: Association of Video Gaming With Cognitive Performance Among Children
Source: JAMA Netw Open. 2022 Oct 24;5(10):e2235721. doi: 10.1001/jamanetworkopen.2022.35721 (PMC9593235; doi:10.1001/jamanetworkopen.2022.35721)
Supplement: Supplement 2. — Retracted Article With Errors Highlighted [file jamanetwopen-e2235721-s002.pdf]

Retracted article with errors highlighted

JAMA Network Open

Original Investigation | Pediatrics

Association of Video Gaming With Cognitive Performance Among Children

Bader Chaarani, PhD; Joseph Ortigara, MS; DeKang Yuan, MS; Hannah Loso, PhD; Alexandra Potter, PhD; Hugh P. Garavan, PhD

Abstract

IMPORTANCE Although most research has linked video gaming to subsequent increases in aggressive behavior in children after accounting for prior aggression, findings have been divided with respect to video gaming's association with cognitive skills.

OBJECTIVE To examine the association between video gaming and cognition in children using data from the Adolescent Brain Cognitive Development (ABCD) study.

DESIGN, SETTING, AND PARTICIPANTS In this case-control study, cognitive performance and blood oxygen level–dependent (BOLD) signal were compared in video gamers (VGs) and non–video gamers (NVGs) during response inhibition and working memory using task-based functional magnetic resonance imaging (fMRI) in a large data set of 9- and 10-year-old children from the ABCD study, with good control of demographic, behavioral, and psychiatric confounding effects. A sample from the baseline assessment of the ABCD 2.0.1 release in 2019 was largely recruited across 21 sites in the US through public, private, and charter elementary schools using a population neuroscience approach to recruitment, aiming to mirror demographic variation in the US population. Children with valid neuroimaging and behavioral data were included. Some exclusions included common MRI contraindications, history of major neurologic disorders, and history of traumatic brain injury.

EXPOSURES Participants completed a self-reported screen time survey including an item asking children to report the time specifically spent on video gaming. All fMRI tasks were performed by all participants.

MAIN OUTCOMES AND MEASURES Video gaming time, cognitive performance, and BOLD signal assessed with n-back and stop signal tasks on fMRI. Collected data were analyzed between October 2019 and October 2020.

RESULTS A total of 2217 children (mean [SD] age, 9.91 [0.62] years; 1399 [63.1%] female) participated in this study. The final sample used in the stop signal task analyses consisted of 1128 NVGs (0 gaming hours per week) and 679 VGs who played at least 21 hours per week. The final sample used in the n-back analyses consisted of 1278 NVGs who had never played video games (0 hours per week of gaming) and 800 VGs who played at least 21 hours per week. The VGs performed better on both fMRI tasks compared with the NVGs. Nonparametric analyses of fMRI data demonstrated a greater BOLD signal in VGs in the precuneus during inhibitory control. During working memory, a smaller BOLD signal was observed in VGs in parts of the occipital cortex and calcarine sulcus and a larger BOLD signal in the cingulate, middle, and frontal gyri and the precuneus.

CONCLUSIONS AND RELEVANCE In this study, compared with NVGs, VGs were found to exhibit better cognitive performance involving response inhibition and working memory as well as altered BOLD signal in key regions of the cortex responsible for visual, attention, and memory processing.

(continued)

Key Points

Question What is the association between video gaming and cognition in children?

Findings As part of the national Adolescent Brain Cognitive Development study and after controlling for confounding effects, results of this case-control study of 2217 children showed enhanced cognitive performance in children who played video games vs those who did not. Clear blood oxygen level–dependent signal differences were associated with video gaming in task-related brain regions during inhibition control and working memory.

Meaning These findings suggest that video gaming may be associated with improved cognitive abilities involving response inhibition and working memory and with alterations in underlying cortical pathways.

+ Invited Commentary

+ Supplemental content

Author affiliations and article information are listed at the end of this article.

Open Access. This is an open access article distributed under the terms of the CC-BY License.

Retracted article with errors highlighted

Abstract (continued)

The findings are consistent with videogaming improving cognitive abilities that involve response inhibition and working memory and altering their underlying cortical pathways.

JAMA Network Open. 2022;5(10):e2235721. doi:10.1001/jamanetworkopen.2022.35721

Introduction

Ask any parent how they feel about their child's videogaming and you'll almost certainly hear concerns about hours spent in a virtual world and the possibility of adverse effects on cognition, mental health, and behavior. A contributing factor to these concerns is the growth of video gaming within the last 20 years. In tandem, the demographic makeup of gamers has also been rapidly changing. In children aged 2 to 17 years, a large 2022 survey in the US showed that 71% play video games, an increase of 4 percentage points since 2018.[1] Given the substantial brain development that occurs during childhood and adolescence, these trends have led researchers to investigate associations between gaming and cognition and mental health. Most psychological and behavioral studies[2] suggest detrimental associations of video gaming, linking it to subsequent increases in depression, violence, and aggressive behavior in children after accounting for prior aggression. However, researchers have been divided with respect to whether playing video games is associated with cognitive skills and brain function. In contrast to the negative associations with mental health, video gaming has been proposed to enhance cognitive flexibility by providing skills that can be transferred to various cognitive tasks relevant for everyday life. One formulation for this broad transfer is that video gaming shares a number of perceptual and attentional demands (such as multiple object tracking, rapid attentional switches, and peripheral vision) with common cognitive tasks and can enhance reaction time (RT), creativity, problem solving, and logic.[3,4]

In a previous review investigating video gaming and cognitive tasks,[3] gaming was found to be associated with attentional benefits including improvements in bottom-up and top-down attention, optimization of attentional resources, integration between attentional and sensorimotor areas, and improvements in selective and peripheral visual attention. Video gamers (VGs) may also benefit from an enhanced visuospatial working memory capacity according to Boot et al,[5] who found that VGs outperformed non-VGs (NVGs) on various visuospatial working memory tasks, such as multiple object tracking, mental rotation, and change detection. Working memory improvements were similarly found after video game training in experimental vs control group research designs.[5-7] This finding is consistent with other studies suggesting that even short video game training paradigms can enhance cognitive control–related functions for long durations, such as reading abilities in dyslexic children[8] and, more particularly, working memory.[3]

Task-based functional magnetic resonance imaging (fMRI) studies[4,9-11] have compared brain activity between VGs and NVGs. When presented with a complex visuomotor task, Granek et al[4] found that VGs exhibited more blood oxygen level–dependent (BOLD) activity in the prefrontal cortex but less overall brain activity compared with NVGs. In 1 study using an fMRI attentional letter detection task, Richlan et al[9] found no significant behavioral performance differences between 14 VGs and 14 NVGs, but VGs showed more brain activation in multiple frontoparietal regions and different activation patterns, suggesting that VGs may recruit different regions of the brain to perform attentional tasks. In the same study,[9] no differences between the 2 groups were observed during a working memory visuospatial task in overall performance (in accuracy or RT) or in brain activation. In a more recent study, Trisolini and colleagues[10] investigated sustained performance between VGs and NVGs in 2 attentional tasks. The results indicated that although VGs displayed significantly stronger performance at the beginning of the task, a substantial decrease in performance was observed over time. By the end of the task, NVGs performed more accurately and quicker. Moreover, in a study[11] investigating the short-term impact of different activities performed during a break before an n-back working memory test in an fMRI scan, 27 young adults who played

video games during the break displayed poorer working memory task performance and less BOLD activity in the supplementary motor area compared with those who had listened to music. However, VGs showed neither performance nor BOLD differences compared with those who spent the break resting. The authors reasoned that the video-gaming demands may have fatigued specific cognitive resources that rely on the supplementary motor area and reduced the ability of VGs to focus attention on the subsequent working memory task.[11] This finding is in contrast with another study[3] that suggested that even short video game training paradigms can enhance cognitive control–related functions, particularly working memory, with the enhancement linked to activity changes in prefrontal areas, such as the dorsolateral prefrontal cortex and the orbitofrontal cortex.

In brief, although several studies have investigated the association between video gaming and cognitive behavior, the neurobiological mechanisms underlying the associations are not well understood because only a handful of neuroimaging studies have addressed this topic. In addition, findings from fMRI studies on video gaming in children and adolescents have not been replicated, which could be in part attributable to the relatively small sample sizes included in the analyses (N<80). In this study, we assess video-gaming associations with cognitive performance and brain activation during response inhibition and working memory using task-based fMRI in a large data set of 9- and 10-year-old children from the Adolescent Brain Cognitive Development (ABCD) study,[12] the largest long-term study of brain development and child health in 21 research sites across the US. We hypothesized, based on the literature, that VGs would perform better on the tasks and have altered cortical activation patterns compared with NVGs in key areas of the brain involved in inhibitory control and working memory.

Methods

This case-control study used data from the baseline assessment of the ABCD study 2.0.1 release in 2019, which recruited a large sample of 9- to 10-year-old children from whom neuroimaging and behavioral data were acquired and quality controlled according to standard operating procedures for the ABCD study consortium.[5] The fMRI paradigms were preprocessed with standard automated pipelines using Analysis of Functional NeuroImages and included the stop signal task (SST) and the n-back task. Children were asked to report how many hours per week they play video games on a computer, console, smart phone, or other devices. Consent (parents) and assent (children) were obtained from all participants. The ABCD study was approved by the appropriate institutional review boards: most ABCD research sites rely on a central Institutional Review Board at the University of California, San Diego for the ethical review and approval of the research protocol, with a few sites obtaining local IRB approval.

Sample

The ABCD sample was largely recruited through public, private, and charter elementary schools. The ABCD study adopted a population neuroscience approach to recruitment[13,14] by using epidemiologically informed procedures to ensure demographic variation in its sample that would mirror the variation in the US population of 9- and 10-year-olds.[15] A probability sampling of schools was conducted within the defined catchment areas of the study's nationally distributed set of 21 recruitment sites in the US. All children in each sampled school were invited to participate after classroom-based presentations, distribution of study materials, and telephone screening for eligibility. Exclusions included common MRI contraindications (such as cardiac pacemakers and defibrillators, internal pacing wires, cochlear and metallic implants, and Swan-Ganz catheters), inability to understand or speak English fluently, uncorrected vision, hearing or sensorimotor impairments, history of major neurologic disorders, gestational age less than 28 weeks, birth weight less than 1200 g, birth complications that resulted in hospitalization for more than 1 month, current diagnosis of schizophrenia, moderate or severe autism spectrum disorder, history of traumatic brain injury, or unwillingness to complete assessments. The ABCD study sample also includes 2105

monozygotic and dizygotic twins. The ABCD study's anonymized data, including all assessment domains, are released annually to the research community. Data on race and ethnicity are not included in the ABCD study data. Information on how to access ABCD study data through the National Institute of Mental Health Data Archive is available on the ABCD study data-sharing webpage.[16]

Screen Time Survey

Participants were administered a screen time survey that asked how much time they spend engaged in different types of screen time on a typical weekday and a typical weekend day. The different screen time categories were as follows: "Watch TV shows or movies?"; "Watch videos (such as YouTube)?"; "Play video games on a computer, console, phone, or other device (Xbox, Play Station, iPad)?"; "Text on a cell phone, tablet, or computer (eg, GChat, Whatsapp, etc.)?"; "Visit social networking sites like Facebook, Twitter, Instagram, etc?"; and "Video chat (Skype, Facetime, etc)?" For each of these activities, the participants responded with how much time they spent per day doing them. They could answer none, less than 30 minutes, 30 minutes, 1 hour, 2 hours, 3 hours, or 4 hours. Answers were mostly none for the texting, social networking, and video chatting categories, as expected for this age range. For each participant, a total weekly video-gaming score was derived as the sum of (video-gaming hours per weekday × 5) + (video-gaming hours per weekend day × 2). A total weekly watching videos score was also derived for each participant. Using the video-gaming score, we defined a group of NVGs who never played video games (0 gaming hours per week) and a group of VGs who played 3 hours per day (21 hours per week) or more. This threshold was selected because it exceeds the American Academy of Pediatrics screen time guidelines,[17] which recommends that video-gaming time be limited to 1 to 2 hours per day for older children.

Demographic Characteristics and Mental Health Measures

The child's age and sex were reported by the parent at the baseline assessment. A trained researcher measured children's height (to the nearest inch) and weight (to the nearest 0.1 lb). Height and weight were assessed 2 times, and means were recorded. Height and weight were converted to body mass index (BMI) age-, sex-, weight-, and height-specific *z* scores (according to the Centers for Disease Control and Prevention age-, sex-, height-, and weight-specific BMI cutoffs[18]). IQ scores were derived from the National Institutes of Health Toolbox cognition battery[19] as the mean of crystalized intelligence and fluid intelligence composite, age-corrected scores. Demographic characteristics were compared between VGs and NVGs using a 2-tailed *t* test except for sex, which was compared using a χ^2 test (**Table 1**).

Mental health symptoms were evaluated using the Child Behavior Checklist (CBCL).[20] Raw scores of behavioral (anxiety, depression, somatic, social, attention, rule breaking, and aggression concerns) and psychiatric categories (*Diagnostic and Statistical Manual of Mental Disorders, Fifth Edition* diagnoses of depression, anxiety, somaticism, attention-deficit hyperactivity disorder, oppositional-defiant disorder, and conduct disorder) were compared between VGs and NVGs using a 2-tailed *t* test.

Task fMRI Acquisition

The ABCD imaging protocol was designed to extend the benefits of high temporal and spatial resolution of imaging protocols of the Human Connectome Project[21] with the multiple scanner systems of participating sites.[22] High spatial and temporal resolution simultaneous multislice and multiband echo-planar imaging task-based fMRIs, with fast integrated distortion correction, were acquired to examine functional activity. For the 3-T scanners (Siemens and GE), the scanning parameters were as follows: matrix, 90 × 90; 60 slices; field of vision, 216 × 216; echo time/repetition time, 800/30 milliseconds; flip angle, 52°; and resolution, 2.4 × 2.4 × 2.4 mm. The fMRI acquisitions (2.4-mm isotropic with repetition time of 800 milliseconds) used multiband echo-planar imaging with slice acceleration factor 6. The order of fMRI tasks was randomized across participants.

Retracted article with errors highlighted

The fMRI preprocessing pipeline included a within-volume head motion estimation and correction and a correction for image distortions. Estimates of task-related activation strength were computed at the individual participant level using a general linear model implemented in Analysis of Functional NeuroImages 3dDeconvolve, with additional nuisance regressors and motion estimates. Hemodynamic response functions were modeled with 2 parameters using a γ-variate basis function plus its temporal derivative.

The SST and n-back task were selected from the ABCD imaging battery to probe inhibitory control and working memory, respectively. Participants practiced the 2 tasks before scanning to ensure they understood the instructions and were familiar with the response collection device. These 2 tasks yield robust neural activation patterns as demonstrated previously.[23] Quality control criteria included excluding participants based on poor image quality, motion, or task performance. The full details of the tasks and fMRI acquisition, preprocessing, and quality control are described in the eMethods in the Supplement and by Hagler et al.[21]

Behavioral Task Performance

The adaptive algorithm used in the SST allowed for calculation of the stop signal RT (SSRT; the time required to inhibit the motor response[23]), which was used as the performance variable in analyses that assessed individual differences in response inhibition ability. The SSRT was computed by subtracting the median stop signal delay of all successful stop trials from the *n*th percentile go RT, where *n* represents the percentage of successful inhibitions (for details on the theoretical underpinnings for this estimation, see the study by Logan and Cowan[24]). To evaluate behavioral task performance in the n-back task, D' (calculated as the *z*-transformed hit rate minus the *z*-transformed false alarm rate) was computed for both the 2-back and 0-back conditions by calculating each participant's hit rate (the proportion of targets for which the participant correctly indicated a match)

Table 1. Demographic Characteristics and Task Performance Measures in NVGs and VGs in the SST and n-Back Samples

Variable	SST sample				n-Back sample			
	NVGs (n = 1128)	VGs (n = 679)	SMD	*P* value	NVGs (n = 1278)	VGs (n = 800)	SMD	*P* value
Video gaming, mean (SD), h/wk	0	25.57 (0.13)	NA	NA	0	25.54 (0.1)	NA	NA
Age, mean (SD), mo	118.95 (0.2)	119.6 (0.25)	2.6	.07[a]	118.9 (0.22)	119.46 (0.28)	2.14	.05[a]
Sex, No. (%)								
Male	288 (24.5)	372 (54.8)	NA	.001[b]	318 (24.9)	434 (54.3)	NA	.001[b]
Female	840 (75.5)	307 (45.2)	NA		960 (75.1)	366 (45.7)	NA	
Watching television or online video streaming, mean (SD), h/wk	11.34 (0.33)	28.54 (1.1)	24.6	<.001[a]	11.71 (0.3)	29.02 (0.51)	43.3	<.001[a]
Combined parental income, mean (SD), income bracket[c]	7.31 (0.06)	6.52 (0.06)	13.1	<.001[a]	7.33 (0.06)	6.49 (0.06)	14	<.001[a]
IQ scores, mean (SD)	89 (2.3)	90 (2.1)	0.95	.10[a]	89 (2.4)	91 (2.2)	1.36	.09[a]
BMI *z* scores, mean (SD)	0.32 (0.05)	0.29 (0.07)	2.12	.10[a]	0.31 (0.06)	0.29 (0.06)	2.1	.10[a]
SSRT, mean (SD), ms	307.22 (9.14)	299.03 (11.46)	0.8	.006[a]	NA	NA	NA	NA
0-back D', mean (SD), ms[d]	NA	NA	NA	NA	2.18 (0.03)	2.33 (0.03)	4.7	.001[a]
2-back D', mean (SD), ms	NA	NA	NA	NA	1.72 (0.02)	1.87 (0.03)	5	.001[a]
Scanner manufacturer, GE/Phillips/Siemens	227/124/777	136/77/466	NA	.18[b]	278/141/859	176/88/536	NA	.21[b]
Handedness, No. (%) left-handed	117 (10.4)	72 (10.6)	NA	.22[b]	137 (10.7)	84 (10.5)	NA	.24[b]

Abbreviations: BMI, body mass index (calculated as weight in kilograms divided by height in meters squared); NA, not applicable; NVGs, non–video gamers; SMD, standardized mean difference; SSRT, stop signal reaction time; SST, stop signal task; VGs, video gamers.

[a] Two-tailed *t* test.

[b] χ^2 Test.

[c] Income brackets are as follows: 1, less than $5000; 2, $5000 to less than $12 000; 3, $12 000 to less than $16 000; 4, $16 000 to less than $25 000; 5, $25 000 to less than $35 000; 6, $35 000 to less than $50 000; 7, $50 000 to less than $75 000; 8, $75 000 to less than $100 000; 9, $100 000 to less than 200 000; and 10, $200 000 or more.

[d] D' was calculated as the *z*-transformed hit rate minus the *z*-transformed false alarm rate.

Retracted article with errors highlighted

and the false alarm rate (the proportion of nontargets for which the participant incorrectly indicated a match or did not respond). The hit and false alarm rates were then *z* transformed.

Participant Inclusion Criteria

Participants were included if they had (1) 2 fMRI runs per task, (2) cortical vertex and subcortical voxel data available at the time of analysis, (3) hemispheric mean BOLD signal within 2 SDs of the sample mean for each task, (4) at least 200 *df* during the 2 scan runs, (5) mean framewise displacement less than 0.9 mm for both runs, (6) met task-specific performance criteria (described in the eMethods in the Supplement), and (7) had complete information on the screen time survey and for all other variables (CBCL, age, sex, scanner serial number, puberty [Peterson et al[25]], and combined parental income).

Statistical Analysis

Collected data were analyzed between October 2019 and October 2020. Analysis of variance and χ^2 tests were used to compare the demographic characteristics between NVGs and VGs. The Permutation Analysis of Linear Models (PALM) general linear model[26] was used to run vertexwise permutation analyses comparing cortical task-specific BOLD signal between NGVs and VGs for correct stop vs correct go and incorrect stop vs correct go conditions of the SST, as well as 0-back vs fixation and 2-back vs fixation conditions of the n-back test, with age (months), sex, scanner serial number, IQ, puberty, and combined parental income included as nuisance covariates. The analyses accommodated the nonindependence of the participants by incorporating information on sibling status as a nested covariate in the model using PALM's exchangeability blocks,[27] which restrict the shuffling to only occur among the observations that share the same family index, that is, within-block only. In addition, performance measures, including SSRT and correct go RT in the SST as well as D' and correct RT in the n-back, were compared between NVGs and VGs using analysis of covariance (with the same covariates as those included in neuroimaging analyses). All statistical tests were 2-sided. False discovery rate–corrected *P* values and statistical maps were considered significant at $P < .05$.

Structural Equation Modeling

To address any confounding effects of time spent watching videos, behavioral problems, or psychiatric disorders on BOLD changes associated with video gaming, we used structural equation modeling to model the association between video gaming and activation in the n-back task and SST, with video watching, behavioral problems, and psychiatric disorders scores included as covariates (**Figure 1**). β Coefficients from the fMRI general linear model (model described in the eMethods in the Supplement) were extracted using MATLAB (MathWorks) for each task and contrast from vertexes showing significant differences between NVGs and VGs in the vertexwise analyses. Mean β coefficients were computed for each contrast and included as the BOLD signal variable in the model. Total behavioral problems and psychiatric disorder scores were calculated from the CBCL[20] as the sum of the scores of all of the problem and psychiatric items, respectively. The model was specified in *R* software, version 4.0.4 (R Foundation for Statistical Computing) using the structural equation

Figure 1. Model Used to Study the Association Between Video Gaming and Activation in the n-Back Task and Stop Signal Task

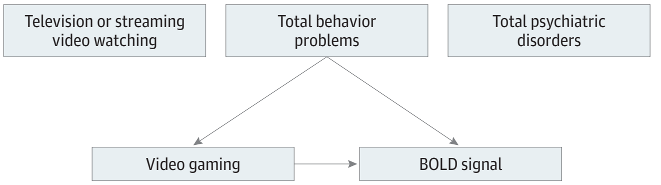

BOLD indicates blood oxygen level dependent.

modeling package lavaan,[28] version 0.6-7. The direct effect of video gaming on BOLD signal (parameter b1) served to check whether any initial association remained significant after controlling for the covariates. This determination was accomplished by letting each covariate predict both video gaming and BOLD signal such that each covariate could have direct effects (represented as b2 and b3) as well as an indirect effect on BOLD signal via video gaming (b1 × b2) (Figure 1). In this regard, video gaming could be interpreted as a mediator of the covariates' effects. The total effect of covariates on the BOLD signal equals b1 × b2 + b3, whereas the covariate-corrected effect of video gaming on the BOLD signal equals b1. The means of the likelihood ratio, defined as measures of the quality of statistical models, were also calculated.

Results

Demographic Characteristics

A total of 2217 children (mean [SD] age, 9.91 [0.62] years; 1399 [63.1%] female) participated in this study (Table 1). The final sample used in the SST analyses consisted of 1128 NVGs (0 gaming hours per week) and 679 VGs who played 21 hours per week or more. The final sample used in the n-back analyses consisted of 1278 NVGs who had never played video games (0 hours per week of gaming) and 800 gamers who played 21 hours per week or more.

The NVG vs VG between-group comparisons showed that groups did not differ on age, BMI, or IQ, but gamers were disproportionately male and had less combined parental income (Table 1). Although consistently higher in VGs, the analyses also revealed that mental health and behavioral scores from the CBCL were not significantly different between NVGs and VGs. In addition, the *t* scores from the CBCL showed that none of the measures in either group was high enough to reach clinical significance.

Individual Behavioral Performance Measures

Performance on the SST was in the anticipated range (mean [SD] SSRT, 302.6 [67.1] milliseconds; mean [SD], go RT, 529.9 [77.4] milliseconds), with a mean (SD) rate of correct inhibitions of 51.4% (0.06%). The distributions for D' were as expected, with children performing better on the 0-back task (mean [SD] D' = 2.51 [0.9] mmilliseconds) than the 2-back task (mean [SD] D' = 2.0 [0.3] milliseconds) ($P < .001$). Analysis of covariance tests compared task performance measures between NVGs and VGs, with age, sex, IQ, scanner, sibling status, and combined parental income included as covariates. Analyses showed that video gaming was associated with improved performance in the SST and n-back tasks (**Figure 2**) and not confounded by sex. In the SST, relative to NVGs, VGs had significantly shorter measures for SSRT and correct go RT measures (mean [SD] time, 299.03 [11.46] vs 307.22 [9.14] milliseconds; $P = .006$). Following a similar pattern, 0-back D' (mean [SD], 2.33 [0.03] vs 2.18 [0.03] milliseconds) and 2-back D' (mean [SD] time, 1.87 [0.03] vs 1.72 [0.02] milliseconds) measures of the n-back task were significantly higher in VGs relative to NVGs ($P = .001$) (Figure 2).

Vertexwise Task fMRI Analyses

In the correct stop vs correct go condition of the SST, vertexwise analyses showed significantly greater BOLD signal in VGs compared with NVGs in the bilateral precuneus (**Figure 3**). No significant differences were observed in the incorrect stop vs correct go condition of the SST.

In the 2-back vs fixation condition of the n-back task, a significantly greater BOLD signal was observed in VGs compared with NVGs in bilateral parts of the dorsal posterior cingulate gyrus, subparietal cortex, middle and superior frontal gyri, and precuneus (Figure 3). Meanwhile, a smaller BOLD signal was observed in VGs in the 2-back vs fixation condition in bilateral parts of the occipital cortex and the calcarine sulcus (Figure 3). The direction, anatomical label, cluster size, and peak vertex number for each cortical region showed significant changes between VGs and NVGs (**Table 2**). Cortical clusters showing these differences in the n-back sample also survive a Bonferroni familywise

Retracted article with errors highlighted

error correction at $P < .05$. Similar patterns of BOLD differences between VGs and NVGs were observed in male and female groups examined separately. No significant differences were observed in the 0-back vs fixation condition of the n-back task.

Structural Equation Modeling

The two structural equation models (for the SST and n-back task) showed good fits with root mean square error of approximation less than 0.04, a comparative fit index greater than 0.9, and Tucker-Lewis Index greater than 0.9. Video watching was positively associated with video gaming for both models (estimates, 0.12 for SST and 0.14 for n-back tasks; $P \leq .001$). However, video watching and total behavioral and psychiatric problems did not have significant direct (b3), indirect (b1 × b2), or total ([b2 × b1] + b3) effects on the BOLD signal in either model. Of importance, the direct effect of video gaming on the BOLD signal remained significant in both models.

Discussion

To date and to our knowledge, this is the largest study to assess the association among video gaming, cognition, and brain function. The behavioral performance findings showed that VGs performed better on both the SST and n-back task compared with NVGs. The fMRI findings demonstrated that VGs show a greater BOLD signal in bilateral parts of the precuneus, using an SST probing inhibitory

Figure 2. Differences of Behavioral Task Performance and Child Behavior Checklist (CBCL) Measures Between Video Gamers and Non-Video Gamers in the Stop Signal Task and n-Back Task

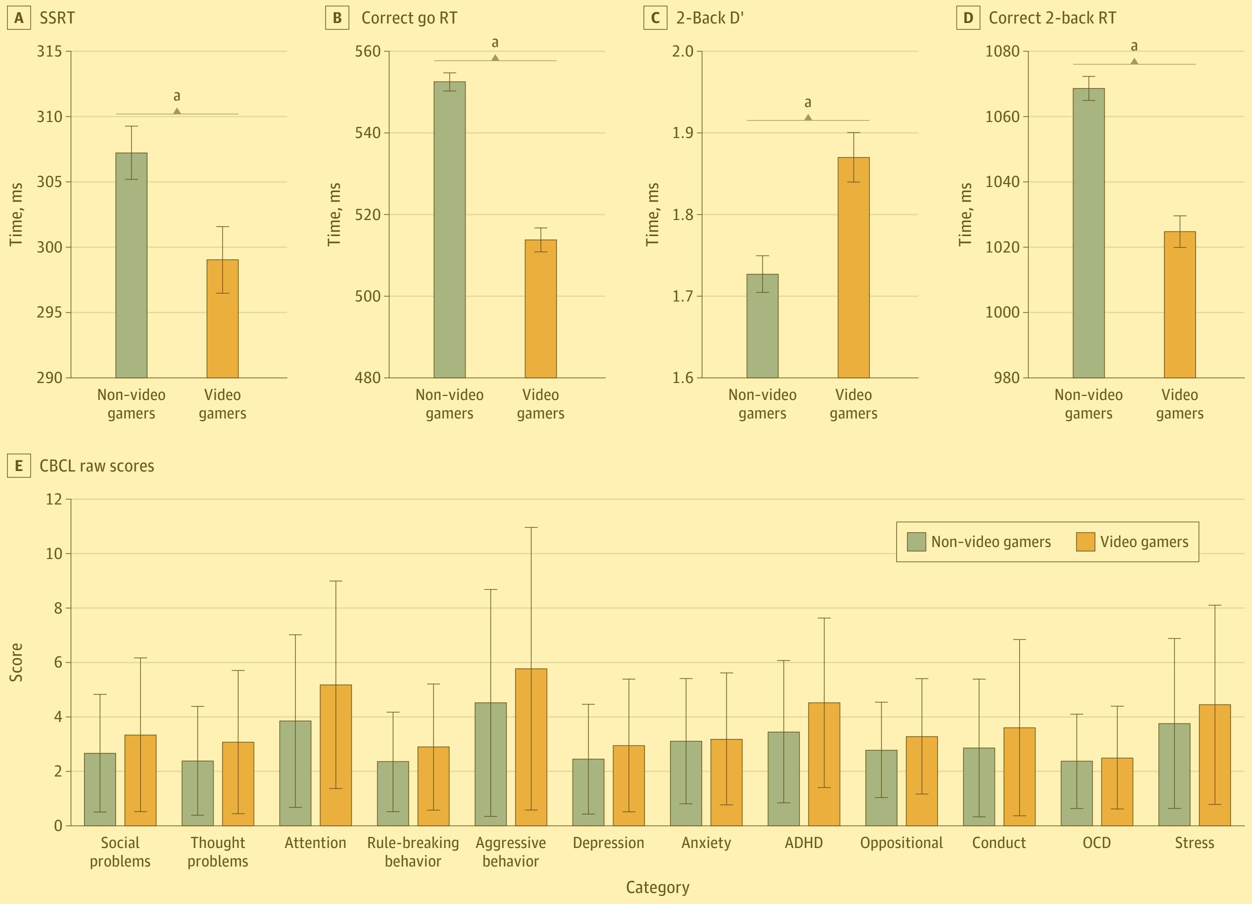

D' was calculated as the z-transformed hit rate minus the z-transformed false alarm rate. Whiskers represent SDs. ADHD indicates attention-deficit/hyperactivity disorder; OCD, obsessive-compulsive disorder; RT, reaction time; and SSRT, stop signal reaction time.

Retracted article with errors highlighted

control. Moreover, results showed a smaller BOLD signal in VGs in parts of the occipital cortex and calcarine sulcus and more activation in cingulate, subparietal, middle, and frontal gyri, and the precuneus during the n-back working memory task. In contrast with psychological and behavioral studies[2] that suggest detrimental associations of video gaming with mental health in children, we did not observe significant differences between VGs and NVGs in our study. However, the higher scores in VGs in every CBCL category leave open the possibility that VGs may be on a trajectory to show larger effects with time and more exposure to video gaming.

The behavioral performance findings in the SST sample are in line with the behavioral findings of the studies by Chisholm et al[29] and Bavelier et al,[30] showing that VGs are less susceptible to attentional distraction and outperform NVGs on both selection-based and response-based

Figure 3. Vertexwise Between-Group Comparisons in Video Gamers vs Non-Video Gamers

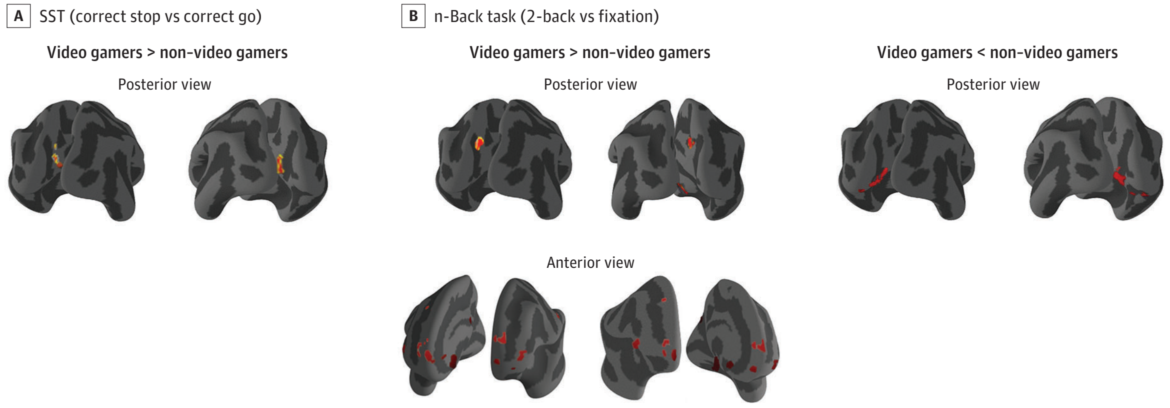

SST indicates stop signal task.

Table 2. Anatomical Label, Cluster Size, and Peak Vertex Number of Cortical Regions Where Significant Changes Were Detected in the SST and n-Back Tasks Between VGs and NVGs

BOLD signal and cortical region	Cluster size (No. of vertexes)	Peak vertex No. (mean space)
**SST/correct stop vs correct go**		
Greater BOLD signal in VGs		
Left precuneus	126	8
Right precuneus	134	30
**n-Back/2-back vs fixation**		
Greater BOLD signal in VGs		
Left posterior cingulate gyrus	48	90
Right posterior cingulate gyrus	64	114
Left precuneus	145	21
Right precuneus	124	6
Left inferior parietal gyrus	98	2304
Right inferior parietal gyrus	73	2284
Left middle frontal gyrus	81	1173
Right middle frontal gyrus	67	1125
Left superior frontal gyrus	168	6563
Right superior frontal gyrus	182	6488
Smaller BOLD signal in VGs		
Left lateral occipital	102	9669
Right lateral occipital	119	9357
Left calcarine sulcus	56	7982
Right calcarine sulcus	68	6992

Abbreviations: BOLD, blood oxygen level dependent; NVGs, non–video gamers; SST, stop signal task; VGs, video gamers.

processes, suggesting that enhanced attentional performance in VGs may be underpinned by a greater capacity to suppress or disregard irrelevant stimuli. However, these results contradict those obtained in previous studies[31,32] that used go/no-go tasks and those showing higher impulsivity levels to be associated with video gaming. These studies[31,32] adopted a different design and outcome measures, included young adult age ranges, and had small sample sizes (n <56). The behavioral performance findings in the n-back task are also in accordance with previous studies showing enhanced visuospatial working memory performance in VGs compared with NVGs[5,33] and in experimental vs control groups after video game training sessions.[5-7,34] In both tasks, the significantly shorter RTs in VGs compared with NVGs while simultaneously performing more accurately may reflect improved cognitive skills acquired through video gaming and not caused by impulsive responding. According to a previous EEG study,[35] earlier latencies in the visual pathways are another feature found in VGs, which may contribute to faster RTs in visual tasks after years of practice. The enhanced cognitive performance on both the SST and n-back task is supported by previous studies showing that VGs outperform NVGs on a range of cognitive tasks[36] (a flanker task, an enumeration task, and 2 attentional blink tasks) and on crystallized and fluid intelligence measures assessed via the Youth National Institutes of Health Toolbox.[37] In addition, supporting our findings, research on video game training in groups of NVGs using action video games (mainly enhancing one's attentional control) demonstrated that video game training consistently led to transferrable improvements in cognitive performance.[38]

The imaging findings showing a greater BOLD signal associated with video gaming during the SST in the precuneus—a brain region involved in a variety of complex functions including attention, cue reactivity, memory, and integration of information—are consistent with previous fMRI studies[3] in children and young adolescents using response inhibition tasks showing more activation in VGs in parietal areas of the cortex, including the precuneus. More broadly, the findings agree with the evidence that VGs display enhanced overall recruitment in a range of attentional control areas during response inhibition tasks.[3] Of interest, in a previous study[39] investigating changes in resting state functional connectivity after video game practice in young participants using a test-retest design, the key finding was increased correlated activity during rest in the precuneus, suggesting that this area exhibits a practice effect associated with the cognitively demanding video games.[39] Advantages for VGs in various attention-demanding tasks have also been reported by Cardoso-Leite et al.[40] Moreover, in line with our findings, an electroencephalography study[41] showed that heavy-use VGs had larger event-related potential amplitudes relative to NVGs in response to numerical targets under high load conditions, suggesting that heavy-use VGs may show greater sensitivity than NVGs to task-relevant stimuli under increased load, which in turn may underpin greater BOLD changes and improved behavioral performance compared with mild-use VGs and NVGs.

Our finding of less activation in VGs in occipital areas while performing better on the n-back task is consistent with a previous fMRI study[33] that used a visuomotor task and showed less activation in occipitoparietal regions in VGs and improved visuomotor task performance; these findings suggest a reduction in visuomotor cognitive costs as a consequence of the video gaming practice. In addition, in line with our results, Granek et al,[4] using an increasingly complex visuomotor fMRI task, observed greater prefrontal activation in 13 VGs who played a mean (SD) of 12.8 (8.6) hours per week during the preceding 3 years compared with 13 NVGs, which the authors related to the increased online control and spatial attention required by VGs for processing complex, visually guided reaching. Similarly, Gorbet and Sergio[42] found that VGs showed less motor-related activity in the cuneus, middle occipital gyrus, and cerebellum, which they explained as an indicator that VGs have greater neural efficiency when conducting visually guided responses. In addition, previous fMRI research has found significantly greater activation related to video gaming in regions associated with working memory, including the subparietal sulcus and the precuneus.[43,44] In a more recent study,[45] changes in BOLD signal in the subparietal lobe, precentral gyrus, and precuneus from before to after training using a video game with a working memory component predicted changes in performance in an untrained working memory task, suggesting a practice-induced plasticity in these regions.

Although video watching is highly confounded with video gaming in our fMRI samples, our models indicate that the response inhibition and working memory effects remained significant when controlling for video watching (in addition to behavioral and psychiatric problems), suggesting that the observed BOLD alterations in the SST and n-back task are more specific to video gaming than video watching. This finding is important because it suggests that children must actively engage with a video's content, as opposed to passively watching a video, to exhibit altered brain activation in key areas of the brain involved in cognition.

Limitations

This study has some limitations. Video games regroup a variety of gaming categories that include action-adventure, shooters, puzzle solving, real-time strategy, simulation, and sports. These specific genres of video games may have different beneficial effects for neurocognitive development[46] because they do not all equally involve interactive (ie, multisensory and motor systems) and executive function processes. In addition, single vs multiplayer games may also have differential impacts on the brain and cognition.[46] Not including the video-gaming genre in our analyses is a limitation of the current study because the screen time survey in the ABCD database does not include additional information on the genre of video games played. Future large studies investigating the association between video gaming and cognition would benefit from including game genre as a moderating variable in analyses. Another limitation of the current study is the use of only cross-sectional study designs, which cannot provide enough evidence to resolve causality or the directionality of the associations among video gaming and other variables. For example, we cannot resolve whether mental health issues or brain function changes precede and drive video gaming or whether video gaming results in mental health symptoms or altered neuroplasticity. Future works benefiting from the longitudinal design of the ABCD study will enable researchers to move beyond association toward causation using causal approaches, such as discordant twin analyses, bayesian causal networks, and machine learning.

Conclusions

Overall, even with consideration of the correlational nature of these cross-sectional data, the current findings are consistent with video gaming being associated with better performance on cognitive tests that involve response inhibition and working memory and altered BOLD signal on these tasks. Thus, although the CBCL scores were elevated in children who play video games for 3 or more hours a day, the results raise the intriguing possibility that video gaming may provide a cognitive training experience with measurable neurocognitive effects. Future ABCD data releases will allow researchers to test for longitudinal effects in which video gaming might improve response inhibition, working memory, and other cognitive functions, as previously suggested in a longitudinal intervention study[34] in which episodic and short-term memory gains were maintained during a 3-month follow-up period. The longitudinal design of the ABCD study will enable within-participant testing for the correlates of accumulated video-gaming practice over the years. By using methods such as cross-lagged correlations or causal inference, researchers can assess whether video gaming is associated with subsequent behavioral issues or neurocognitive development in adolescents.

ARTICLE INFORMATION
Accepted for Publication: August 20, 2022.

Published: October 24, 2022. doi:10.1001/jamanetworkopen.2022.35721

Open Access: This is an open access article distributed under the terms of the CC-BY License. © 2022 Chaarani B et al. *JAMA Network Open*.

Retracted article with errors highlighted

Corresponding Author: Bader Chaarani, PhD, Department of Psychiatry, University of Vermont, 1 S Prospect St, Burlington, VT 05405 (melmarsr@uvm.edu).

Author Affiliations: Department of Psychiatry, University of Vermont, Burlington.

Author Contributions: Drs Chaarani and Garavan had full access to all of the data in the study and take responsibility for the integrity of the data and the accuracy of the data analysis.

Concept and design: Chaarani, Garavan.

Acquisition, analysis, or interpretation of data: All authors.

Drafting of the manuscript: Chaarani, Ortigara, Garavan.

Critical revision of the manuscript for important intellectual content: Chaarani, Yuan, Loso, Potter, Garavan.

Statistical analysis: Chaarani, Yuan, Loso, Garavan.

Obtained funding: Chaarani, Potter, Garavan.

Administrative, technical, or material support: Chaarani, Ortigara, Potter.

Supervision: Chaarani, Garavan.

Conflict of Interest Disclosures: Dr Potter reported receiving grants from the National Institutes of Health during the conduct of the study. No other disclosures were reported.

Additional Information: Data used in the preparation of this article were obtained from the Adolescent Brain Cognitive Development (ABCD) Study (https://abcdstudy.org) held in the National Institute of Mental Health Data Archive. Computations were performed on the Vermont Advanced Computing Core supported in part by award OAC-1827314 from the National Science Foundation.

REFERENCES

1. The NPD Group. Video games. Accessed September 22, 2022. https://www.npd.com/industry-expertise/video-games

2. Prescott AT, Sargent JD, Hull JG. Metaanalysis of the relationship between violent video game play and physical aggression over time. *Proc Natl Acad Sci U S A*. 2018;115(40):9882-9888. doi:10.1073/pnas.1611617114

3. Palaus M, Marron EM, Viejo-Sobera R, Redolar-Ripoll D. Neural basis of video gaming: a systematic review. *Front Hum Neurosci*. 2017;11:248. doi:10.3389/fnhum.2017.00248

4. Granek JA, Gorbet DJ, Sergio LE. Extensive video-game experience alters cortical networks for complex visuomotor transformations. *Cortex*. 2010;46(9):1165-1177. doi:10.1016/j.cortex.2009.10.009

5. Boot WR, Kramer AF, Simons DJ, Fabiani M, Gratton G. The effects of video game playing on attention, memory, and executive control. *Acta Psychol (Amst)*. 2008;129(3):387-398. doi:10.1016/j.actpsy.2008.09.005

6. Green CS, Bavelier D. Effect of action video games on the spatial distribution of visuospatial attention. *J Exp Psychol Hum Percept Perform*. 2006;32(6):1465-1478. doi:10.1037/0096-1523.32.6.1465

7. Blacker KJ, Curby KM, Klobusicky E, Chein JM. Effects of action video game training on visual working memory. *J Exp Psychol Hum Percept Perform*. 2014;40(5):1992-2004. doi:10.1037/a0037556

8. Franceschini S, Gori S, Ruffino M, Viola S, Molteni M, Facoetti A. Action video games make dyslexic children read better. *Curr Biol*. 2013;23(6):462-466. doi:10.1016/j.cub.2013.01.044

9. Richlan F, Schubert J, Mayer R, Hutzler F, Kronbichler M. Action video gaming and the brain: fMRI effects without behavioral effects in visual and verbal cognitive tasks. *Brain Behav*. 2017;8(1):e00877. doi:10.1002/brb3.877

10. Trisolini DC, Petilli MA, Daini R. Is action video gaming related to sustained attention of adolescents? *Q J Exp Psychol (Hove)*. 2018;71(5):1033-1039. doi:10.1080/17470218.2017.1310912

11. Liu S, Kaufmann C, Labadie C, et al. Short-term effects of video gaming on brain response during working memory performance. *PLoS One*. 2019;14(10):e0223666. doi:10.1371/journal.pone.0223666

12. Casey BJ, Cannonier T, Conley MI, et al; ABCD Imaging Acquisition Workgroup. The Adolescent Brain Cognitive Development (ABCD) study: imaging acquisition across 21 sites. *Dev Cogn Neurosci*. 2018;32:43-54. doi:10.1016/j.dcn.2018.03.001

13. Falk EB, Hyde LW, Mitchell C, et al. What is a representative brain? neuroscience meets population science. *Proc Natl Acad Sci U S A*. 2013;110(44):17615-17622. doi:10.1073/pnas.1310134110

14. Paus T. *Population Neuroscience*. Springer-Verlag; 2013. doi:10.1007/978-3-642-36450-1

15. Garavan H, Bartsch H, Conway K, et al. Recruiting the ABCD sample: design considerations and procedures. *Dev Cogn Neurosci*. 2018;32:16-22. doi:10.1016/j.dcn.2018.04.004

16. 2022 ABCD Study. Data sharing. Accessed August 31, 2022. https://abcdstudy.org/scientists/data-sharing/

17. American Academy of Pediatrics. Media and children. Accessed August 31, 2022. https://www.aap.org/en/patient-care/media-and-children/

18. Kuczmarski RJ, Ogden CL, Guo SS, et al. 2000 CDC growth charts for the United States: methods and development. *Vital Health Stat 11*. 2002;(246):1-190.

19. National Institutes of Health Toolbox. Accessed August 31, 2022. https://www.healthmeasures.net/explore-measurement-systems/nih-toolbox

20. Achenbach TM. Child Behavior Checklist. In: Kreutzer JS, DeLuca J, Caplan B, eds. *Encyclopedia of Clinical Neuropsychology*. Springer; 2011:546-552. doi:10.1007/978-0-387-79948-3_1529

21. Hagler DJ Jr, Hatton S, Cornejo MD, et al. Image processing and analysis methods for the Adolescent Brain Cognitive Development Study. *Neuroimage*. 2019;202:116091. doi:10.1016/j.neuroimage.2019.116091

22. Chaarani B, Hahn S, Allgaier N, et al; ABCD Consortium. Baseline brain function in the preadolescents of the ABCD Study. *Nat Neurosci*. 2021;24(8):1176-1186. doi:10.1038/s41593-021-00867-9

23. Logan GD, Schachar RJ, Tannock R. Impulsivity and inhibitory control. *Psychol Sci*. 1997;8:60-64. doi:10.1111/j.1467-9280.1997.tb00545.x

24. Logan GD, Cowan WB. On the ability to inhibit thought and action: a theory of an act of control. *Psychol Rev*. 1984;91:295. doi:10.1037/0033-295X.91.3.295

25. Petersen AC, Crockett L, Richards M, Boxer A. A self-report measure of pubertal status: reliability, validity, and initial norms. *J Youth Adolesc*. 1988;17(2):117-133. doi:10.1007/BF01537962

26. Winkler AM, Ridgway GR, Webster MA, Smith SM, Nichols TE. Permutation inference for the general linear model. *Neuroimage*. 2014;92:381-397. doi:10.1016/j.neuroimage.2014.01.060

27. Winkler AM, Webster MA, Vidaurre D, Nichols TE, Smith SM. Multi-level block permutation. *Neuroimage*. 2015;123:253-268. doi:10.1016/j.neuroimage.2015.05.092

28. Rosseel Y. lavaan: an R package for structural equation modeling. *J Stat Softw*. 2012;48:1-36. doi:10.18637/jss.v048.i02

29. Chisholm JD, Hickey C, Theeuwes J, Kingstone A. Reduced attentional capture in action video game players. *Atten Percept Psychophys*. 2010;72(3):667-671. doi:10.3758/APP.72.3.667

30. Bavelier D, Achtman RL, Mani M, Föcker J. Neural bases of selective attention in action video game players. *Vision Res*. 2012;61:132-143. doi:10.1016/j.visres.2011.08.007

31. Azizi E, Stainer MJ, Abel LA. Is experience in multi-genre video game playing accompanied by impulsivity? *Acta Psychol (Amst)*. 2018;190:78-84. doi:10.1016/j.actpsy.2018.07.006

32. Luijten M, Meerkerk GJ, Franken IHA, van de Wetering BJM, Schoenmakers TM. An fMRI study of cognitive control in problem gamers. *Psychiatry Res*. 2015;231(3):262-268. doi:10.1016/j.pscychresns.2015.01.004

33. Lee H, Voss MW, Prakash RS, et al. Videogame training strategy-induced change in brain function during a complex visuomotor task. *Behav Brain Res*. 2012;232(2):348-357. doi:10.1016/j.bbr.2012.03.043

34. Toril P, Reales JM, Mayas J, Ballesteros S. Video game training enhances visuospatial working memory and episodic memory in older adults. *Front Hum Neurosci*. 2016;10:206. doi:10.3389/fnhum.2016.00206

35. Latham AJ, Patston LLM, Tippett LJ. The virtual brain: 30 years of video-game play and cognitive abilities. *Front Psychol*. 2013;4:629. doi:10.3389/fpsyg.2013.00629

36. Green CS, Bavelier D. Action video game modifies visual selective attention. *Nature*. 2003;423(6939):534-537. doi:10.1038/nature01647

37. Walsh JJ, Barnes JD, Tremblay MS, Chaput JP. Associations between duration and type of electronic screen use and cognition in US children. *Comput Human Behav*. 2020;108:106312. doi:10.1016/j.chb.2020.106312

38. Green CS, Bavelier D. Learning, attentional control, and action video games. *Curr Biol*. 2012;22(6):R197-R206. doi:10.1016/j.cub.2012.02.012

39. Martínez K, Solana AB, Burgaleta M, et al. Changes in resting-state functionally connected parietofrontal networks after videogame practice. *Hum Brain Mapp*. 2013;34(12):3143-3157. doi:10.1002/hbm.22129

40. Cardoso-Leite P, Kludt R, Vignola G, Ma WJ, Green C, Bavelier D. Technology consumption and cognitive control: contrasting action video game experience with media multitasking. *Atten Percept Psychophys*. 2016;78(1):218-241. doi:10.3758/s13414-015-0988-0

41. Mishra J, Zinni M, Bavelier D, Hillyard SA. Neural basis of superior performance of action videogame players in an attention-demanding task. *J Neurosci*. 2011;31(3):992-998. doi:10.1523/JNEUROSCI.4834-10.2011

42. Gorbet DJ, Sergio LE. Move faster, think later: women who play action video games have quicker visually-guided responses with later onset visuomotor-related brain activity. *PLoS One*. 2018;13(1):e0189110. doi:10.1371/journal.pone.0189110

43. Dahlin E, Neely AS, Larsson A, Bäckman L, Nyberg L. Transfer of learning after updating training mediated by the striatum. *Science*. 2008;320(5882):1510-1512. doi:10.1126/science.1155466

44. Koenigs M, Barbey AK, Postle BR, Grafman J. Superior parietal cortex is critical for the manipulation of information in working memory. *J Neurosci*. 2009;29(47):14980-14986. doi:10.1523/JNEUROSCI.3706-09.2009

45. Nikolaidis A, Voss MW, Lee H, Vo LT, Kramer AF. Parietal plasticity after training with a complex video game is associated with individual differences in improvements in an untrained working memory task. *Front Hum Neurosci*. 2014;8:169. doi:10.3389/fnhum.2014.00169

46. Brilliant T D, Nouchi R, Kawashima R. Does video gaming have impacts on the brain: evidence from a systematic review. *Brain Sci*. 2019;9(10):251. doi:10.3390/brainsci9100251

SUPPLEMENT.

eMethods. Supplemental Methods
